# Erythrocytosis as an indicator of disease progression of small cell lung cancer: A case report

**DOI:** 10.1016/j.rmcr.2025.102240

**Published:** 2025-05-25

**Authors:** Takuya Tanaka, Yutaka Takahara, Ryudai Abe, Nagae Sumito, Yoko Ishige, Ikuyo Shionoya, Kouichi Yamamura, Masafumi Nojiri, Masaharu Iguchi

**Affiliations:** Department of Respiratory Medicine, Kanazawa Medical University, 1-1 Daigaku, Kahoku-gun, Uchinada-machi, Ishikawa, 920-0293, Japan

**Keywords:** Small cell lung cancer, Paraneoplastic syndrome, Polycythemia, Erythropoietin

## Abstract

This case involves a 62-year-old male diagnosed with extensive-stage small cell lung cancer (ES-SCLC) originating from the right lower lobe with brain metastases. The patient underwent stereotactic radiosurgery (SRS) and received four cycles of combination therapy with cisplatin, etoposide, and durvalumab, followed by maintenance therapy with durvalumab to achieve disease control. On day 156 after treatment initiation, hemoglobin (Hb) levels increased to 16.9 g/dL, indicating polycythemia. Concurrently, new brain metastatic lesions were identified, and serum erythropoietin (EPO) levels were markedly elevated at 406 mIU/mL. Whole-brain radiation therapy (WBRT) was initiated, resulting in a subsequent reduction in hemoglobin levels and improvement in polycythemia. This report describes a rare case of secondary polycythemia associated with ES-SCLC. Notably, WBRT appeared to contribute to the normalization of serum EPO levels and resolution of polycythemia. This report elucidates the clinical course and relevance of this condition based on a literature review.

## 1 Introduction

Small cell lung cancer (SCLC) frequently presents with paraneoplastic syndromes [[1], [2], [3], [4], [5]]. However, polycythemia is a rare paraneoplastic manifestation [6]. In this article, we report a case of SCLC with brain metastases associated with elevated erythropoietin (EPO) levels and polycythemia.

In this patient, polycythemia was observed in conjunction with disease progression and improved following radiotherapy for metastatic brain tumors. This finding suggests that disease progression may contribute to the development of polycythemia and that radiotherapy may play a role in its management. We report this case, along with a literature review, to highlight this rare association and explore the potential mechanisms underlying these findings.

## 2 Case report

A 62-year-old man with hypertension and a smoking history (5–6 cigarettes/day since age 22) was diagnosed with extensive-stage SCLC in the right lower lobe with brain metastases. He received four cycles of cisplatin, etoposide, and durvalumab, followed by durvalumab maintenance therapy. The disease remained stable; however, on Day 156, his hemoglobin (Hb) increased to 16.9 g/dL, indicating erythrocytosis. On Day 191, the Hb level further increased to 19.0 g/dL, indicating worsening polycythemia, and the serum EPO level increased to 406 mIU/mL. On Day 202, the patient experienced vomiting and a brief loss of consciousness lasting approximately 1 min, prompting emergency transportation to our hospital and subsequent admission.

The vital signs upon admission were as follows: temperature, 36.3 °C; pulse, 96 beats/min (regular); and blood pressure, 128/71 mm Hg. The SpO_2_ was 97 % on room air. Facial flushing was also observed. The patient was alert and oriented, with no signs of visual field defects, neck stiffness, paralysis, or speech disturbance.

Laboratory results revealed an erythrocyte count of 5.3 million/μL and a Hb level of 19.0 g/dL, indicating polycythemia. The level of the tumor marker, pro-gastrin-releasing peptide, was elevated at 605 pg/mL.

**Fig. 1:**
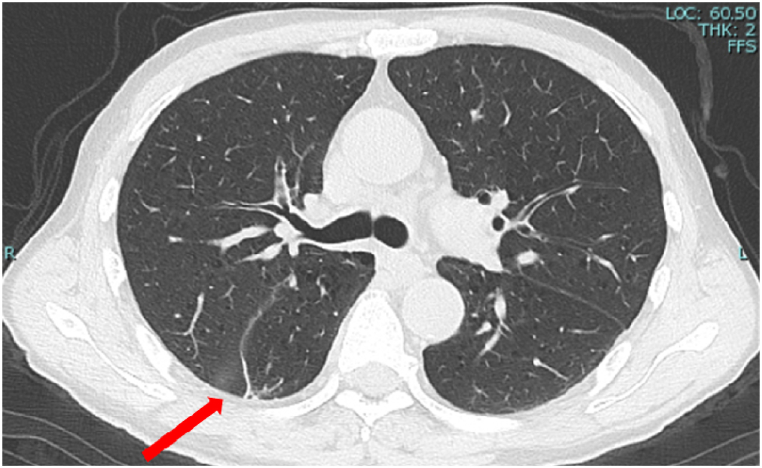
Chest CT on admission. A small scar-like nodular shadow can be seen on the pleural side of the right lower lobe (arrow).

**Fig. 2:**
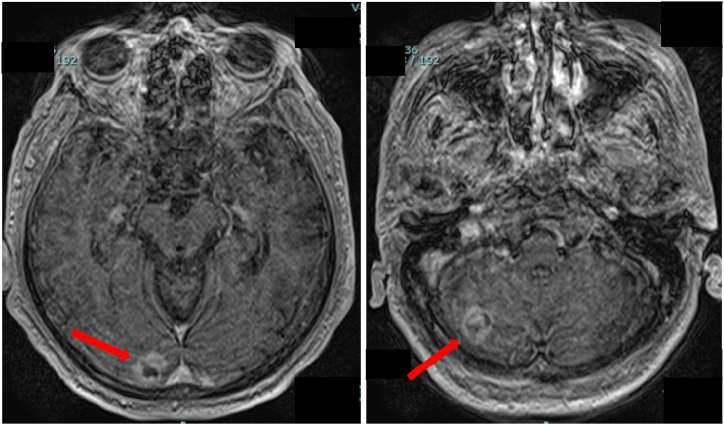
Contrast-enhanced MRI of the head. Multiple brain metastases are observed in the cerebral and cerebellar hemispheres (arrows) MRI, magnetic resonance imaging.

**Fig. 3:**
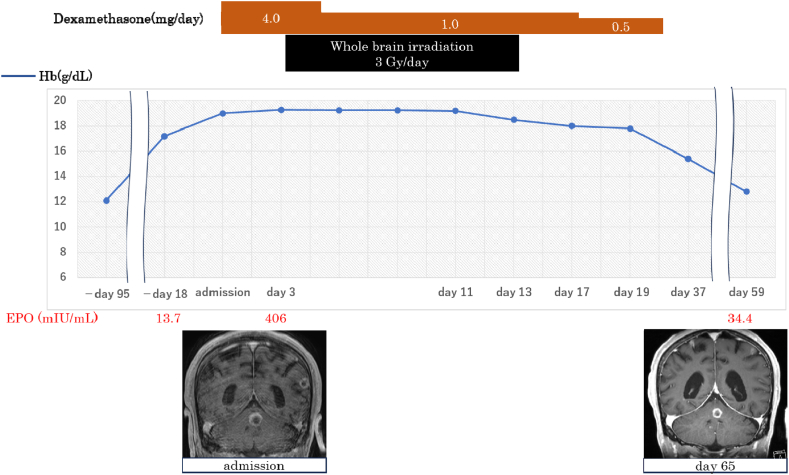
Clinical course of the patient. Clinical data, laboratory results, radiological findings, and treatment are presented.

Upon admission, plain chest and abdominal CT scans (Fig. 1) revealed a small scar-like nodular shadow on the pleural side of the right lower lobe, with no evidence of tumor recurrence in other organs, including the kidneys, adrenal glands, and liver. Contrast-enhanced brain magnetic resonance imaging confirmed the presence of multiple brain metastases in both cerebral hemispheres and the cerebellum (Fig. 2). JAK2 V617F mutation testing performed during hospitalization returned negative results. The course of treatment is illustrated in Fig. 3. Dexamethasone (4 mg/day) and whole-brain irradiation were administered to prevent the progression of brain metastases. Thereafter, subjective symptoms such as vomiting and loss of consciousness improved, Hb levels decreased, and the patient was discharged on the 28th day.

## 3 Discussion

In this paper, we report a case of SCLC complicated by polycythemia. The findings suggest that radiation therapy normalizes serum EPO levels and improves polycythemia. “Erythrocytosis” refers to a condition in which Hb or hematocrit (Hct) levels increase relative to or above the normal range [7,8].

Secondary erythrocytosis (SE) is a heterogeneous group of disorders primarily caused by the abnormal activation of EPO due to inadequate tissue oxygenation. Conditions such as chronic obstructive pulmonary disease and cyanotic heart disease with a right-to-left shunt are recognized causes of hypoxia-induced acquired erythrocytosis [9].

In this patient, continuous SpO_2_ monitoring during both outpatient visits and hospitalization revealed no evidence of hypoxemia, suggesting a low likelihood of hypoxia-induced polycythemia. Additionally, the patient had no history of diuretic use or renal transplantation [10], both of which are known causes of non-hypoxic secondary erythrocytosis.

Autonomous EPO production is a reported cause of secondary polycythemia in a variety of malignant tumors, including renal cell carcinoma, meningioma, and hepatocellular carcinoma [6,9,[11], [12], [13], [14], [15], [16]].

Considering the localization of EPO-producing tumors, reports include 179 cases in the kidney, 64 cases in the liver, 50 cases in the central nervous system, 25 cases in the uterus, 11 cases in the adrenal glands, 7 cases in the ovaries, 3 cases in the lungs, and 1 case in the thymus [6]. Furthermore, the literature describes only one reported case of an EPO-producing tumor caused by lung cancer [17]. These findings suggest that EPO-producing tumors originating from lung cancer are extremely rare.

**Fig. 4:**
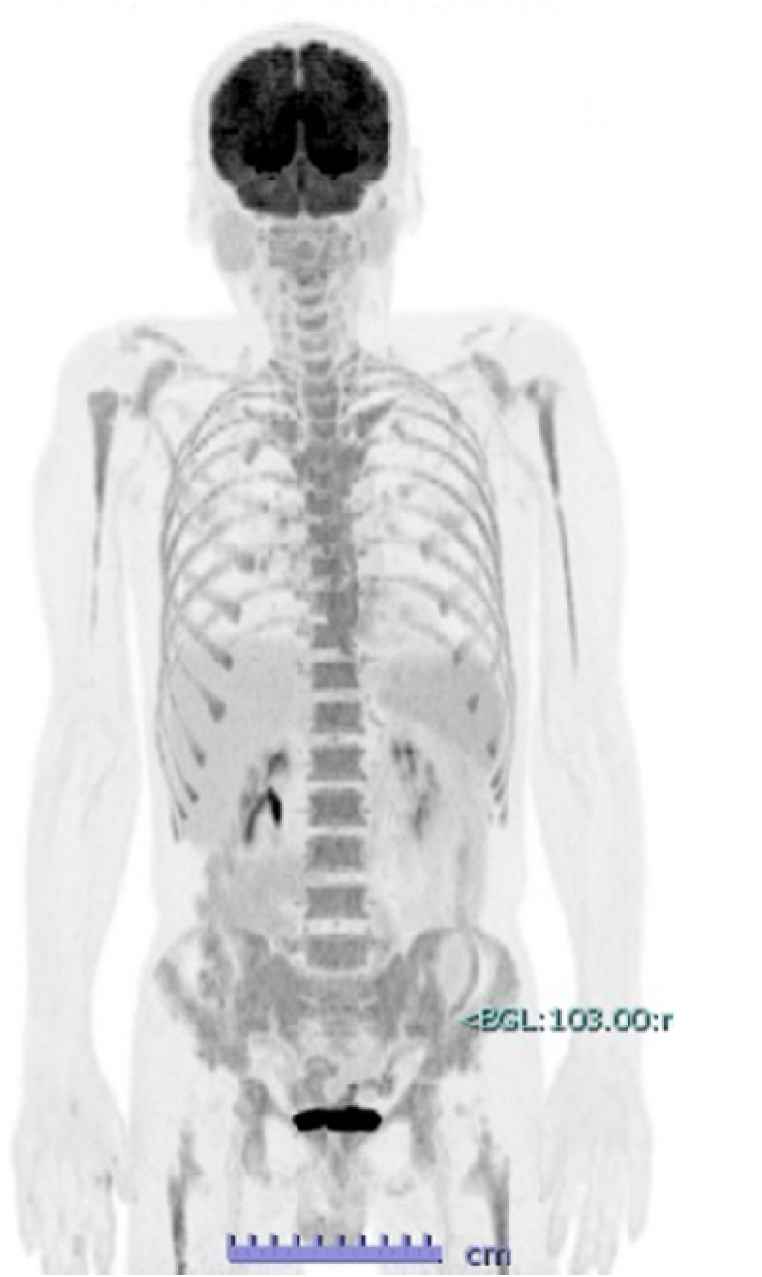
PET-CT performed two months prior to admission. No abnormal FDG uptake was observed in the primary lesion or other organs.

In this case, the onset of polycythemia coincided with the emergence of brain metastases. A PET-CT performed two months prior to admission (Fig. 4) showed no abnormal uptake in other organs, and chest and abdominal CT scans upon admission revealed no tumors in the kidneys, adrenal glands, liver, or other organs. Following radiotherapy for the brain metastases, both polycythemia and elevated EPO levels improved rapidly, leading to a diagnosis of erythrocytosis due to EPO production from the metastatic brain tumor.

In general, the management of acquired erythrocytosis requires addressing the underlying cause [11]. In this patient, early radiation therapy for metastatic brain tumors improved polycythemia without thrombotic complications.

In this case, corticosteroid therapy was administered concurrently with whole-brain radiotherapy (WBRT) for brain metastases; therefore, we cannot entirely rule out the possibility that steroids contributed to the suppression of erythrocytosis. However, glucocorticoids are generally recognized to promote the maintenance and proliferation of erythroid progenitor cells [18], and a direct causal relationship between corticosteroid administration and the observed decrease in Hb levels in this case is not clearly established.

Furthermore, while we cannot entirely rule out the possibility that the treatment of immune-related adverse events (irAEs) associated with immune checkpoint inhibitors (ICIs) contributed to the improvement of polycythemia, it is important to note that irAEs more commonly involve anemia or thrombocytopenia [19]. To date, there have been no reports linking ICIs to erythrocytosis. Therefore, a clear causal relationship between ICI therapy and erythrocytosis has not been established.

Additionally, in this case, erythrocytosis did not recur even after tapering and discontinuing corticosteroids. Based on this clinical course, it is more likely that local tumor control through WBRT for metastatic brain lesions contributed to the improvement in erythrocytosis and elevated serum EPO levels.

Paraneoplastic polycythemia in SCLC is extremely rare. In this case, erythrocytosis appeared to be associated with tumor activity and resolved following local treatment, suggesting a potential link between disease progression and increased erythropoiesis. Polycythemia is a known risk factor for thromboembolic events [20]. In cases where tumor-related erythrocytosis is suspected, early diagnosis and careful clinical monitoring are crucial to prevent serious complications. When polycythemia is observed in patients with SCLC, clinicians should consider not only the possibility of hypoxia or hematologic disorders but also evaluate for recurrence, metastasis, and the potential for ectopic EPO production.

## CRediT authorship contribution statement

**Takuya Tanaka:** Writing – original draft, Conceptualization. **Yutaka Takahara:** Writing – original draft, Data curation. **Ryudai Abe:** Data curation. **Nagae Sumito:** Data curation. **Yoko Ishige:** Data curation. **Ikuyo Shionoya:** Writing – original draft, Data curation. **Kouichi Yamamura:** Data curation. **Masafumi Nojiri:** Data curation. **Masaharu Iguchi:** Writing – original draft, Data curation.

## Funding

This research received no specific grant from any funding agency in the public, commercial, or not-for-profit sectors.

## Declaration of competing interest

The authors declare that they have no known competing financial interests or personal relationships that could have appeared to influence the work reported in this paper.

## References

[1] Patel A.M., Davila D.G., Peters S.G. (1993). Paraneoplastic syndromes associated with lung cancer. Mayo Clin. Proc..

[2] Hauber H.P. (2011). Paraneoplastic syndromes in lung cancer. Pneumologie.

[3] Gandhi L., Johnson B.E. (2006). Paraneoplastic syndromes associated with small cell lung cancer. J. Natl. Compr. Cancer Netw..

[4] Honnorat J., Antoine J.C. (2007). Paraneoplastic neurological syndromes. Orphanet J. Rare Dis..

[5] Titulaer M.J., Soffietti R., Dalmau J. (2011). Screening for tumours in paraneoplastic syndromes: report of an EFNS task force. Eur. J. Neurol..

[6] Hammond D., Winnick S. (1974). Paraneoplastic erythrocytosis and ectopic erythropoietins. Ann. N. Y. Acad. Sci..

[7] Pearson T.C. (1991). Apparent polycythaemia. Blood Rev..

[8] Fairbanks V.F., Tefferi A. (2000). Normal ranges for packed cell volume and hemoglobin concentration in adults: relevance to ‘apparent polycythemia’. Eur. J. Haematol..

[9] Gangat N., Szuber N., Tefferi A. (2023). JAK2 unmutated erythrocytosis: 2023 Update on diagnosis and management. Am. J. Hematol..

[10] Yoon J.S., Kang H., Jekarl D.W., Lee S.E., Oh E.J. (2024). Diagnostic performance of serum erythropoietin to discriminate polycythemia vera from secondary erythrocytosis through established subnormal limits diagnostic performance of serum erythropoietin to discriminate polycythemia vera from secondary erythrocytosis through established subnormal limits. Diagnostics.

[11] Sakisaka S., Watanabe M., Tateishi H. (1993). Erythropoietin production in hepatocellular carcinoma cells associated with polycythemia: immunohistochemical evidence. Hepatology (Baltim., Md.).

[12] Matsuyama M., Yamazaki O., Horii K. (2000). Erythrocytosis caused by an erythropoietin-producing hepatocellular carcinoma. J Surg. Oncol..

[13] Nielsen O.J., Jespersen F.F., Hilden M. (1988). Erythropoietin-induced secondary polycythemia in a patient with a renal cell carcinoma. A case report. APMIS.

[14] Noguchi Y., Goto T., Yufu Y. (1999). Gene expression of erythropoietin in renal cell carcinoma. Intern. Med..

[15] Matsuo M., Koga S., Kanetake H. (2003). EPO-producing gastric carcinoma in a hemodialysis patient. Am. J. Kidney Dis..

[16] Munakata W., Ohashi K., Sakaguchi K. (2010). Erythrocytosis caused by erythropoietin-producing thymic carcinoma. Int. J. Clin. Oncol..

[17] Miyoshi I., Uemura Y., Nakai T., Daibata M., Ly M., Saintigny P. (2009). Erythropoietin-producing lung cancer. Intern. Med..

[18] Hattangadi S.M., Wong P., Zhang L., Flygare J., Lodish H.F. (2011). From stem cell to red cell: regulation of erythropoiesis at multiple levels by multiple proteins, RNAs, and chromatin modifications from stem cell to red cell: regulation of erythropoiesis at multiple levels by multiple proteins, RNAs, and chromatin modifications. Blood.

[19] Li N., Feng Y., Chen X., Li Y., Zhang C., Yin Y. (2023). Hematologic and lymphatic system toxicities associated with immune checkpoint inhibitors: a real-world study. Front. Pharmacol..

[20] Griesshammer M., Kiladjian J.J., Besses C. (2019). Thromboembolic events in polycythemia vera Thromboembolic events in polycythemia vera. Ann. Hematol..

